# Exercise-altered fluid shear stress regulates vascular endothelial inflammation via Piezo1

**DOI:** 10.3389/fphys.2026.1875135

**Published:** 2026-09-10

**Authors:** Shuo Yang, Song Wang

**Affiliations:** 1 School of Sports Medicine, Wuhan Sports University, Wuhan, China; 2 Sports Immunology Research Center, Wuhan Sports University, Wuhan, China; 3 The College of Sports Science and Technology of Wuhan Sports University, Wuhan, China

**Keywords:** endothelial inflammation, exercise, fluid shear stress, mechanotransduction, Piezo1

## Abstract

Exercise, as the primary physiological mechanism for modulating blood fluid shear stress (FSS), plays a crucial role in regulating vascular endothelial function by mediating mechanical signal transduction through the mechanosensor Piezo1. Recent studies have demonstrated that physiological laminar shear stress is essential for maintaining vascular homeostasis, whereas disturbed flow, induced by various factors, leads to endothelial inflammation. This review summarizes the changes in hemodynamic characteristics mediated by different exercises, provides an overview of the structure of the Piezo1, and its physiological functions in endothelial cells. Focuses on analyzing the mechanisms through which fluid shear stress regulates vascular endothelial inflammation via Piezo1. This review aims to comprehensively analyze the potential mechanisms of exercise-induced hemodynamic changes in the protection against vascular diseases and to provide a theoretical basis for Piezo1-targeted therapies in cardiovascular diseases and related chronic inflammation.

## 1 Introduction

The global burden of cardiovascular disease (CVD) has doubled over the past three decades and is projected to reach 1.14 billion cases by 2050. Early prevention and intervention have the potential to substantially reduce this burden (Naeem et al., 2026). Endothelial inflammation is closely associated with endothelial dysfunction, plaque formation, and other pathological events, drives the onset and progression of CVD (Henein et al., 2022), Regular exercise effectively reduce the risk of CVD (Lear et al., 2017), Exercise elevates mean shear stress while reducing flow recirculation and the oscillatory shear index (OSI) at the mouse aortic arch; this hemodynamic shift subsequently activates endothelial SCD1, thereby alleviating endothelial inflammation and endoplasmic reticulum (ER) stress to preserve vascular endothelial homeostasis (Cavallero et al., 2024). Fluid shear stress (FSS) is the frictional force generated between blood flowing per unit area and the endothelium of blood vessels (Malek et al., 1999). It includes both anterograde and retrograde components (Chatlaong et al., 2025). The hemodynamic patterns within blood vessels are complex. Pulsatile laminar shear stress (Hsieh et al., 2009), unidirectional laminar shear stress (Wang et al., 2016a) enhance the anti-inflammatory and antioxidant capacities of endothelial cells through multiple signaling pathways, thereby contributing to the maintenance of a healthy endothelial phenotype. However, in areas such as bifurcation and tortuosity of blood vessels, disturbed flow often forms. Disturbed flow is typically characterized by low time-averaged wall shear stress (TAWSS)or high oscillatory shear stress (OSS), reflecting reduced mean wall shear stress and periodic flow reversal over the cardiac cycle. This complex hemodynamic feature is a typical blood flow feature of atherosclerosis (AS) susceptible areas (Bacigalupi et al., 2024). It triggers the development of AS inflammation through mechanotransduction pathways on endothelial cells, promoting endothelial inflammatory cascade reactions and dysfunction (Chen et al., 2024). Exercise generates distinct shear stress patterns (Tinken et al., 2009). Moderate intensity aerobic exercise increases laminar shear stress (LSS) with a predominantly antegrade direction (Coovert et al., 2017), thereby activating endothelial nitric oxide synthase (eNOS) (Garcia et al., 2022) and inducing an anti-inflammatory, anti-thrombotic phenotype in endothelial cells (ECs). FSS is a key exercise-induced mechanical stimulus that directly acts on vascular ECs.

Piezo1 is a mechanosensitive cation channel that responds to distinct FSS patterns by converting them into intracellular biological signals. Piezo1 is highly expressed in vascular ECs and vascular smooth muscle cells (VSMCs), mediates Ca^2+^ influx, activates multiple downstream signaling pathways, and participates in regulating ECs’ inflammatory response, oxidative stress, and ECs-VSMCs crosstalk. Piezo1 activation exerts opposing biological effects depending on the pattern of blood flow shear stress (Davis et al., 2023). Piezo1 activation upregulates genes that promote anti-inflammatory and antioxidant effects in the endothelium (Nunez et al., 2023), thereby maintaining vascular homeostasis. However, it can also mediate pro-inflammatory responses, exacerbate endothelial cell damage, and contribute to the pathogenesis of vascular diseases such as AS and venous insufficiency. Comprehensive reviews have systematically summarized the general principles of vascular mechanotransduction (Davis et al., 2023; Lim and Harraz, 2024; Power et al., 2024) as well as the important role of Piezo1 in the regulation of inflammation. However, how exercise shaping different FSS patterns, and how these patterns differentially regulate endothelial inflammation via Piezo1 have not been systematically integrated. The present review integrates these two aspects to address this gap. It is important to note that direct evidence for exercise-induced Piezo1 activation in humans is still lacking. Accordingly, the present review offers a mechanistic framework that will require validation in future human studies.

## 2 Exercise modality determines fluid shear stress profiles

### 2.1 The biomechanical microenvironment of vascular endothelial cells

Vascular ECs are constantly exposed to a complex mechanical microenvironment during physiological and pathological processes. In the straight segments of human large arteries, blood flow follows a smooth, unidirectional laminar pattern, this pattern generates laminar shear stress on ECs, aligning them in the direction of flow, strengthening both adherens and tight junctions between ECs by upregulating VE-cadherin and claudin-5 (McQueen and Warboys, 2023), and promoting the release of vasodilators and increasing vascular compliance, LSS is essential for maintaining endothelial homeostasis and function (Nawara et al., 2025). However, due to factors such as pulsatility, complex vascular geometry, and vessel elasticity, disturbed flow commonly occurs in regions such as bifurcations, vascular curvatures, and stenoses within the human vasculature (Liu et al., 2024). The direction of disturbed flow is highly variable, generating OSS and low shear stress at local sites on the vascular wall (Wang et al., 2025), This abnormal mechanical microenvironment induces ECs to exhibit a pro-inflammatory phenotype (Thacher et al., 2010; Wang et al., 2022a) and oxidative stress (Davies, 2009) in ECs, thereby contributing to the initiation and progression of AS through multiple ways, including leukocyte adhesion and migration, thrombosis, and endothelial-to-mesenchymal transition (EndMT), etc (Wang et al., 2023a).

The mechanical forces experienced by the vascular wall include three types of exogenous stimuli: FSS, hydrostatic pressure and cyclic stretch (Hahn and Schwartz, 2009; Liu et al., 2025), as well as endogenous forces such as cytoskeletal tension (Humphrey and Schwartz, 2021). These mechanical signals are sensed by endothelial mechanosensors at the cell membrane or cell-cell junctions, such as PECAM-1 and VE-cadherin. Through the cytoskeleton and the LINC (Linker of Nucleoskeleton and Cytoskeleton) complex, these forces are rapidly transmitted to the nuclear envelope, where they regulate nuclear morphology, lamin organization, chromatin architecture, and gene transcription (Mannion and Holmgren, 2023).

According to Poiseuille’s law, FSS is proportional to blood viscosity and flow velocity, but inversely proportional to the third power of vessel diameter (Beech, 2018). In humans, under physiological conditions, FSS intensity varies across different vascular beds. In the arterial system, it typically ranges from 10 to 70 dyn/cm² (Malek et al., 1999), generally exceeding 15 dyn/cm², and exhibits a unidirectional laminar flow pattern. In contrast, the venous wall experiences a much lower FSS, approximately 1–6 dyn/cm² (Malek et al., 1999). Exercise, pathological conditions, and other factors alter the direction and magnitude of FSS. By regulating downstream signaling pathways and gene expression, FSS directs distinct endothelial cell phenotypes, ultimately shaping vascular structure and function.

### 2.2 Exercise regulates blood flow shear stress

**Figure 1:**
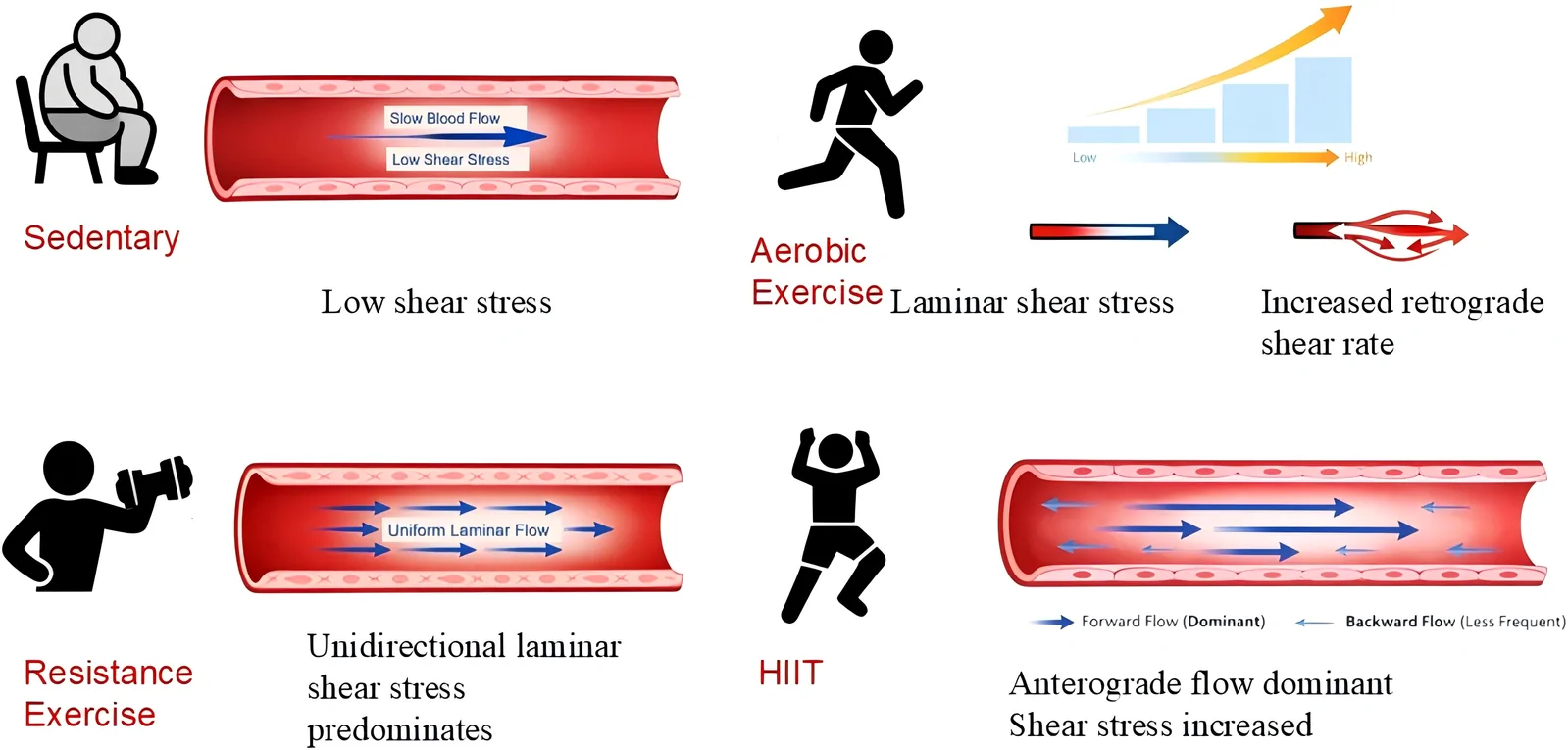
The impact of different exercises on hemodynamic characteristics. Sedentary, aerobic exercise, resistance exercise, and HIIT induce distinct hemodynamic profiles. Sedentary and obese individuals have slower blood flow velocity and lower wall shear stress. Aerobic exercise produces bidirectional blood flow with increasing retrograde flow at higher intensities. Resistance exercise predominantly generates unidirectional laminar flow. HIIT elevates shear stress and induces retrograde flow, yet anterograde flow remains dominant.

Sedentary lifestyle and obesity are risk factors for CVD, and the underlying mechanisms may involve reduced FSS. In humans, prolonged sitting for 6 hours significantly reduced mean blood flow velocity, shear rate, blood flow and FSS in peripheral arteries, thereby affecting vascular function (Restaino et al., 2015). In obese individuals, increased red blood cell aggregation and reduced blood flow velocity also contribute to a decrease in wall shear stress (Wiewiora et al., 2013). Low shear stress levels below 4 dyn/cm² in human arteries are associated with inflammatory status in ECs (Wang et al., 2022a). Exercise increases FSS intensity. The resulting changes in FSS amplitude and frequency can regulate nitric oxide (NO) and reactive oxygen species (ROS) levels in ECs (Wang et al., 2018). However, the hemodynamic responses elicited by different exercise modalities vary substantially (**Figure 1**).

Aerobic exercise upregulates vascular endothelial growth factor and eNOS expression in ECs by enhancing LSS, thereby increasing NO production and bioavailability, promoting vasodilation, and maintaining vascular tone and blood pressure stability (Kim et al., 2024). LSS concurrently activates autophagy-related genes (Gur, 2020), suppresses pro-inflammatory cytokines and adhesion molecules, and preserves vascular endothelial function. Even short-term regular aerobic exercise can induce protective vascular adaptations through LSS, including anti-inflammatory and antioxidant effects (Robinson et al., 2017). However, long-term moderate-intensity aerobic exercise further induces vascular remodeling, characterized by luminal dilation and wall thinning (Green et al., 2017). Long-term exercise enhances vascular tolerance to high FSS, which may partly explain the improved vascular adaptability observed after training (Maufroy et al., 2026a). Human studies have quantified the FSS induced by different exercise intensities. In a controlled *in vitro* model using human umbilical vein endothelial cells (HUVECs), adopted a fixed experimental gradient based on previous *in vivo* measurements, reporting that low-, moderate-, and high-intensity aerobic exercise elicit FSS of approximately 35, 50, and 70 dyn/cm², respectively (Conde et al., 2024). Subsequently, the same group established a standardized protocol utilizing exercise-induced FSS values derived from *in vivo* human measurements (Conde et al., 2025). This protocol offers a standardized FSS testing operation process that stratifies exercise intensity based on maximal oxygen uptake and blood lactate thresholds. By applying the Womersley model, they estimated *in vivo* arterial FSS values across three conditions: rest (18 dyn/cm²), low-intensity exercise (35 dyn/cm²), and high-intensity exercise (60 dyn/cm²). This framework provides a highly universal physiological reference range that simultaneously accounts for the stability of *in vitro* experiments, thereby establishing a physiologically relevant foundation for subsequent *in vitro* mechanobiological simulations. Hemodynamic changes induced by exercise intensity exceeding the physiological range expose ECs to abnormal mechanical stress, potentially leading to vascular damage. The higher the intensity of acute aerobic exercise, the greater the reduction in blood flow-mediated vasodilation post-exercise (Birk et al., 2013). Prolonged high-intensity and high-load training may contribute to plaque formation (Cheng et al., 2023). Individuals with a lifelong high cumulative exercise volume exhibit a significantly higher prevalence of coronary artery calcification and atherosclerotic plaques compared to low-volume counterparts. Notably, the predominance of calcified plaques in this population may reflect adaptive remodeling of the vessel wall may reflect vascular adaptation to long-term increases in FSS (Aengevaeren et al., 2017).

Blood flow direction exhibits pronounced phasic variations throughout the cardiac cycle. In vascular physiology, anterograde flow refers to blood movement in the physiological direction, whereas retrograde flow denotes movement opposite to the expected direction. During systole, the forward ejection of blood from the heart toward the peripheral circulation constitutes anterograde flow. Conversely, retrograde flow represents a transient reversal of blood movement, typically occurring in early diastole. These bidirectional flow components generate distinct temporal patterns of shear stress, thereby exerting differential mechanical stimuli on the vascular endothelium (Green, 2009; Ade et al., 2012). There are differences in the hemodynamic characteristics induced by resistance exercise and aerobic exercise. Aerobic exercise is characterized by sustained high blood flow with both anterograde and retrograde directions (Coovert et al., 2018), As exercise intensity increases, the intensity and frequency of retrograde flow rise (Green et al., 2005), may resulting in a higher retrograde shear rate. Resistance exercise significantly increases blood flow, FSS, and anterograde shear rate in the active limb, where the blood flow pattern remains predominately a unidirectional laminar flow (Thijssen et al., 2009; Thomas et al., 2020). Both resistance and aerobic exercise may activate the Piezo1 channel by increasing unidirectional shear stress, and contributes to the redistribution of blood flow during exercise and helps maintain vascular homeostasis (Beech, 2018). This suggests that the protective mechanisms of these two exercise modalities on the vasculature may be similar.

There is currently no consensus on the hemodynamic characteristics induced by high-intensity interval training (HIIT). In human studies, acute HIIT has been shown to produces a higher oscillatory shear index (OSI) than continuous exercise, this may be attributed to reduced anterograde shear accompanied by preserved retrograde shear, potentially resulting in a more oscillatory shear environment (Lyall et al., 2019). However, chronic low-volume HIIT has been reported to improve shear profiles by increasing anterograde shear and reducing both retrograde shear and OSI, with greater reductions in retrograde shear compared with continuous moderate-intensity exercise (Ghardashi Afousi et al., 2018). In terms of FSS intensity, a human exercise intervention with matched hemodynamic load revealed that, HIIT induced a significantly higher capillary peak shear stress (18.15 Pa) than MICT (7.45 Pa), demonstrating that exercise intensity directly determines FSS magnitude. The higher FSS generated by HIIT was associated with increased pericyte coverage and enhanced microvascular adaptation (Maufroy et al., 2026b). Hemodynamic responses to exercise are vascular bed-specific. Hemodynamics differ at the carotid artery, the common carotid artery maintains unidirectional laminar flow without retrograde shear or oscillatory flow across all exercise modalities tested (Montalvo et al., 2022). Different blood flow patterns activate distinct signaling networks, thereby determining whether endothelial cell fate leans toward homeostasis or inflammation (Lim and Harraz, 2024).

Compared with human studies, direct measurements of FSS parameters in animal experiments remain limited. Numerous animal studies have demonstrated the protective effect of exercise on vascular endothelial function. Twelve weeks of voluntary wheel running in mice improved endothelial-dependent dilation, inhibited oxidative stress, enhanced eNOS activity, and promoted endothelial adaptation (Durrant et al., 2009). Similar responses have been observed following acute exercise. Mice following a single bout of 50-minutes treadmill running at approximately 80% of maximal oxygen uptake exhibited increased eNOS phosphorylation and endothelial NO production in large arteries compared with sedentary controls (Zhang et al., 2009). The increase in endothelial eNOS phosphorylation may be attributed to the exercise-induced elevation in fluid shear stress (Laughlin et al., 2008). Furthermore, long-term voluntary wheel running in mice has been shown to increase endothelial mitochondrial content. *In vitro* studies demonstrated that physiologically relevant LSS of 20 dyne/cm^2^, mimicking the hemodynamic forces induced by exercise, upregulates genes involved in mitochondrial biogenesis and increases mitochondrial DNA content and mass in human aortic endothelial cells. Collectively, these findings suggest that long-term exercise induced increases in blood flow and shear stress may contribute to enhanced endothelial mitochondrial biogenesis (Kim et al., 2014).

In recent years, more direct evidence has emerged. Using four-dimensional computational fluid dynamics, a study demonstrated that two weeks of voluntary wheel running increased TAWSS in the mouse aortic arch, and reduced flow recirculation and OSI, protected the vascular endothelial (Cavallero et al., 2024). Another study in hypertensive rats observed similar hemodynamic adaptations, showing that 12 weeks of moderate-intensity treadmill training elevated TAWSS in the aortic arch and reduced OSI (Wang et al., 2024). These findings suggest that long-term exercise may shift aortic hemodynamic patterns from oscillatory flow toward laminar, high shear stress conditions, thereby exerting vascular protective effects.

Exercise is a key physiological modulator of FSS. Several endothelial structures are crucial for FSS transduction, including endothelial junctional complex PECAM-1-VEGFR2-VE-cadherin, the cytoskeleton, lipid rafts, and caveolae (Power et al., 2024), G-protein-coupled receptors (Chachisvilis et al., 2006), ion channels (Delmas and Coste, 2013), integrins (Huijing et al., 2002), glycocalyx (Yao et al., 2007), and others, which participation in sensing and transducing mechanical forces (Tzima et al., 2006; Augustin and Koh, 2024). Endothelial mechanotransduction is a complex process involving the synergistic interplay of multiple receptors in both force sensing and downstream signaling, with extensive crosstalk among these mechanosensitive mechanisms (Lim and Harraz, 2024). Among these sensors, the glycocalyx acts as a key initial sensor, transmitting flow mediated FSS to intracellular signals (Power et al., 2024). It may function upstream of the mechanosensitive ion channel Piezo1, triggering Ca^2+^ influx and thereby playing an important role in vascular inflammation, remodeling, and tone regulation.

## 3 Exercise-induced activation of Piezo1

Piezo1 is a mechanosensitive ion channel that enables endothelial cells to sense blood flow and transduce mechanical stimuli into intracellular signals. Rode et al. first demostrated Piezo1 as an exercise sensor in 2017 (Rode et al., 2017). Although direct evidence from humans is still limited, a growing body of animal research supports a causal link between Piezo1 and exercise-induced vascular adaptation. Rode et al. demonstrated that during whole-body exercise, elevated blood flow increases fluid shear stress, which activates endothelial Piezo1 to induce vasoconstriction in specific vascular beds. This mechanism drives blood flow redistribution and vascular homeostasis, ultimately regulating blood pressure and exercise capacity. Consistently, endothelial-specific knockout of Piezo1 markedly impairs exercise capacity in mice.

Subsequent research has uncovered the downstream pathways linking endothelial Piezo1 signaling to exercise capacity. Endothelial Piezo1 deficiency impairs exercise endurance in mice, driven by muscle microvascular endothelial cell apoptosis and capillary rarefaction. During exercise, heightened blood flow and FSS stimulate endothelial Piezo1, which sustains eNOS expression to boost NO production. Consequently, NO downregulates TSP2, safeguarding capillary density and microcirculatory homeostasis—a pivotal axis determining endurance exercise capacity (Bartoli et al., 2022).

Beyond its role in physical performance, Piezo1 mediates exercise-induced improvements in vascular mechanical properties. Voluntary wheel running restores femoral artery compliance in aged mice, counteracting age-related stiffening. Using a partial femoral ligation model that induces sustained low shear stress, the study found that low FSS significantly upregulates Piezo1 expression in endothelial cells, smooth muscle cells, and macrophages—paralleling the stiffening phenotype observed in aging arteries. Consistently, PIEZO1 mRNA expression is elevated in severely stenotic femoral arteries from patients with peripheral arterial disease, positioning Piezo1 as a potential therapeutic target. These findings suggest that exercise-induced hemodynamic adaptations may regulate Piezo1-dependent pathways, although direct evidence for this causal relationship remains limited (Zhao et al., 2025).

Beyond its established role in endothelial cells, Piezo1 mediates the beneficial impacts of physical activity across other tissues; notably, its mechanical activation extends well beyond FSS to encompass other physical forces such as stretch and tension. In skeletal muscle stromal cells, Piezo1 senses mechanical stress and initiates exercise-induced inflammation and tissue adaptation (Langston et al., 2026). In cardiomyocytes, treadmill exercise has been shown to upregulate Piezo1 expression and attenuate apoptosis via the p38MAPK-YAP1 pathway, improving cardiac function after myocardial infarction. Notably, Piezo1 signaling in cardiomyocytes exhibits bidirectionality: physiological mechanical stimulation (exercise or moderate stretch) confers cardioprotection, whereas excessive pharmacological activation (high-dose Yoda1) promotes apoptosis (Duan et al., 2026).

In myoblasts, *in vitro* studies utilizing microfluidic organ-on-a-chip platforms demonstrate that mechanical stimulation mimicking exercise activates the Piezo1 channel. High-intensity compression or tension induces myoblasts damage and detachment, while low-intensity stimulation upregulates Piezo1 and synergizes with Talin1 to promote cytoskeletal remodeling and myofiber repair (Yin et al., 2026). Given that Piezo1 also mediates shear-induced signaling in endothelial cells, this cytoskeletal organizing effect may have relevance to vascular remodeling. Animal studies have shown that vascular deformation resulting from skeletal muscle contraction during physical activity can also activate endothelial Piezo1, suggesting that Piezo1 functions as a polymodal mechanosensor capable of integrating multiple types of mechanical stimuli to regulate vascular function (Jia et al., 2025). Future studies should combine conditional Piezo1 knockout models with well-controlled exercise regimens to further establish causal relationships.

## 4 Piezo1: a core mechanosensor for shear stress sensing in endothelial cells

### 4.1 Activation mechanism of endothelial Piezo1

Given that exercise serves as the primary physiological stimulus that altering hemodynamic forces, understanding how exercise-induced shear stress activates Piezo1 is fundamental to deciphering its vascular protective effects. Piezo1 is a homotrimeric protein composed of 2,547 amino acids (Beech and Kalli, 2019), featuring a characteristic three-bladed propeller-like structure. It consists of three functional domains: the N-terminal blade domain responsible for mechanosensation, the transduction module which includes the beam and central anchor, and the C-terminal pore domain involved in ion conduction. This structural architecture enables Piezo1 to respond to mechanical forces through a lever-like gating mechanism (Tang et al., 2022). Piezo1 can be activated by various mechanical forces, including hydrostatic pressure, membrane stretch, and FSS (Gudipaty et al., 2017). Local hemodynamic changes induce membrane stretch, which opens Piezo1 channels and triggers Ca^2+^ influx. This Ca^2+^ signaling plays a critical role in regulating key biological processes, such as ECs inflammation and AS (Albarrán-Juárez et al., 2018), oxidative stress (Zhang et al., 2024), apoptosis (Liang et al., 2019), and autophagy (Mao et al., 2023). *In vitro* studies using murine endothelial cells have demonstrated that Piezo1 expression is upregulated within the physiological range of 0–30 dyn/cm^2^, but significantly decreases *in vitro* when FSS exceeds 75 dyn/cm^2^, a level considered pathologically high, this inverse correlation between high shear stress and Piezo1 expression was also observed in human intracranial aneurysm samples (Lu et al., 2025).

### 4.2 Physiological function of endothelial Piezo1

As a key mechanosensitive receptor, Piezo1 orchestrates multiple vascular functions, including angiogenesis, vascular development, maintenance of endothelial integrity and barrier function in mature vessels, as well as regulation of blood flow. Furthermore, it engages in crosstalk with other mechanoreceptors such as integrin (Aglialoro et al., 2020), TRPV4 (Allerkamp et al., 2025) and PECAM-1 (Chuntharpursat-Bon et al., 2023). Piezo1 continuously senses endothelial FSS to maintain NO-mediated vasodilation; consequently, Piezo1 knockdown significantly suppresses shear stress-induced Ca^2+^ influx and AKT/eNOS phosphorylation (Wang et al., 2016b). In mice, Piezo1 deletion causes embryonic lethality because ECs fail to respond to FSS, leading to disruption of vascular development (Li et al., 2014). Furthermore, Piezo1, as an upstream sensor of FSS, can activate downstream disintegrin and metalloproteinases (ADAMs). Activated ADAMs regulate ECs inflammation, proliferation, and migration by cleaving and remodeling cell junction proteins (Pabst et al., 2026). In ECs, Piezo1 activation mediates the influx of Ca^2+^ and Na^+^ (Li et al., 2014), reducing the transmembrane potential and inducing EC depolarization. This depolarizing signal is subsequently transmitted to VSMCs via myoendothelial junctions, leading to vasoconstriction (Rode et al., 2017). During systemic exercise, increased blood flow generates FSS, which activates endothelial Piezo1 ion channels, and contributes to vascular tone regulation in specific vascular beds, such as mesenteric circulation, and thereby contributing to blood flow redistribution and vascular homeostasis. Endothelial Piezo1 knockout mice exhibit significantly impaired exercise capacity (Rode et al., 2017), which may be related to the role of Piezo1 in regulating blood redistribution during exercise (Beech, 2018).

Piezo1 function is influenced by the type and size of the vascular bed. In different vessels or under different blood flow patterns, Piezo1 triggers distinct downstream signaling pathways (Albarrán-Juárez et al., 2018). Endothelial Piezo1 channels regulate multiple downstream pathways and play a role in chronic inflammation and injury of the cardiovascular system (Shinge et al., 2022). Multiple studies have demonstrated that Piezo1 activates the NF-κB signaling pathway and upregulates the expression of inflammatory factors in response to disturbed flow (Sun et al., 2025). Furthermore, mechanical activation of Piezo1 disrupts the endothelial barrier, serving as an early event in leukocyte extravasation and promoting chemotaxis and adhesion of inflammatory cells (Wang et al., 2022b). On the other hand, Piezo1 also regulates anti-inflammatory transcriptional pathways, maintains endothelial homeostasis, and protects against endothelial ferroptosis and inflammation (Miao et al., 2025). Piezo1 is a potential target for intervening in mechanical stress-related chronic inflammation (Wang et al., 2023b).

## 5 Piezo1 in shear stress-driven endothelial inflammation

**Figure 2:**
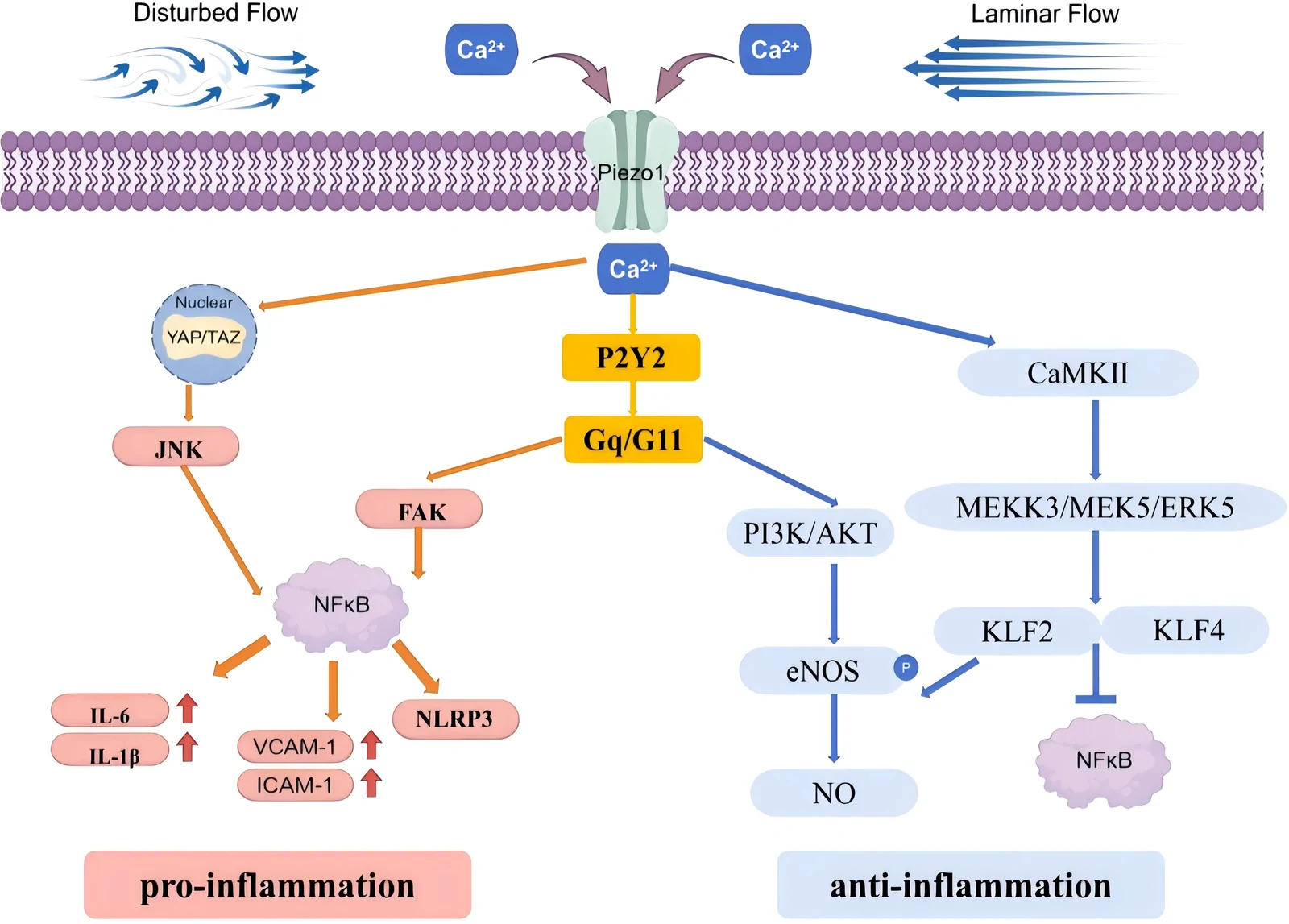
The regulatory mechanism of Piezo1 on endothelial inflammation. Under physiological laminar flow, activation of Piezo1 leads to Ca2+ influx, which triggers two parallel anti-inflammatory pathways: the Piezo1-PI3K-AKT-eNOS-NO pathway and the Piezo1-CaMKII‑MEKK3/MEK5/ERK5-KLF2/KLF4 pathway. In contrast, disturbed flow activates Piezo1 and sets off a pro-inflammatory cascade. This involves YAP/TAZ nuclear translocation, NF-κB activation, and upregulation of adhesion molecules, chemokines, and pro-inflammatory cytokines, ultimately leading to endothelial dysfunction and inflammation.

Given that exercise modulates both the magnitude and profile of shear stress in an intensity- and modality-dependent manner, the distinct endothelial inflammatory responses induced by different flow patterns have important implications for exercise physiology. Exercise-induced elevation of laminar shear stress is widely accepted to activate endothelial Piezo1, leading to Ca^2+^ influx and subsequent eNOS/NO activation, which relaxes vascular smooth muscle cells, thereby promoting vasodilation and increasing blood flow (Rode et al., 2017; Beech, 2018; Maufroy et al., 2026a). A study demonstrated that endothelial-specific Piezo1 knockout in mice directly reduces eNOS expression and NO bioavailability. Consequently, upregulation of the endothelial cell apoptosis-inducing factor TSP2 promotes endothelial cell apoptosis, resulting in pericyte loss and capillary rarefaction, which ultimately impairs motor function. This pathway confirms that Piezo1 is essential for exercise-induced cardiovascular adaptation and functional improvement (Bartoli et al., 2022). Similarly, a study demonstrated that the high FSS generated by HIIT increases pericyte coverage and enhances microvascular stability in humans (Maufroy et al., 2026b). However, disturbed blood flow is a complex hemodynamic pattern that generates both low shear stress (typically <4 dyn/cm²) and OSS(characterized by periodic blood flow reversal). Due to factors such as prolonged sitting and vascular structure, endothelial cells may be exposed to low shear stress or oscillatory shear stress. In contrast to laminar flow, disturbed flow promotes Piezo1-dependent pro-inflammatory signaling. Currently, direct evidence regarding the activation of Piezo1 by different types of exercise remains limited. Nevertheless, accumulating studies have revealed that different FSS patterns can modulate endothelial inflammation through Piezo1-mediated regulation of multiple signaling pathways (**Figure 2**).

### 5.1 Disturbed flow activates endothelial inflammation via Piezo1

#### 5.1.1 Piezo1-YAP/TAZ signaling

Disturbed flow triggers Piezo1-dependent Ca^2+^ influx in endothelial cells, which promotes the nuclear translocation of Yes-associated protein (YAP). This process may be achieved through two synergistic mechanisms: First, the LINC complex serves as a physical bridge between the cytoskeleton and the nuclear envelope, providing the structural foundation for transmitting mechanical signals to the nucleus and regulating YAP/TAZ activity (Takata and Matsumura, 2022). Second, mechanical force-induced increases in nuclear membrane tension have been shown to deform the nuclear pore complex and widen its pore size in U2OS osteosarcoma cells (Francis et al., 2025). While direct evidence in endothelial cells is currently lacking, this mechanism is proposed to occur similarly in the vascular endothelium, given the conservation of the nuclear envelope machinery and the fact that endothelial cells are constantly exposed to hemodynamic forces. Activation of this YAP/TAZ pathway upregulates the expression of key pro-inflammatory mediators, including JNK, TNF-α, NF-κB, and VCAM-1, thereby exacerbating endothelial injury. Studies have shown that Piezo1 expression is upregulated in mouse atherosclerotic carotid plaques, and the associated ECs inflammatory response is closely linked to the Piezo1–YAP/TAZ signaling pathway. Knockdown of Piezo1 significantly inhibits YAP/TAZ activation and the subsequent expression of downstream inflammatory factors (Yang et al., 2022). YAP/TAZ is a core effector of the Hippo pathway and a key mediator of mechanotransduction. Its activation enhances JNK signaling and promotes NF-κB nuclear translocation. Consequently, it upregulates the expression of adhesion molecules, including VCAM-1 and ICAM-1, as well as pro-inflammatory factors such as IL-6 and IL-1β, thereby inducing an endothelial inflammatory response. *In vitro* studies have shown that inhibition of JNK phosphorylation significantly attenuates NF-κB nuclear translocation and its downstream pro-inflammatory effects (Zhou et al., 2017).

#### 5.1.2 Piezo1-NF-κB/NLRP3 signaling

Disturbed flow activates Piezo1 channels on ECs, leading to Ca^2+^ influx and subsequent ATP release into the extracellular space. ATP then binds to P2Y2 receptors, activating Gq/G11 signaling, which in turn triggers integrin activation. This is followed by FAK-dependent activation of the NF-κB pathway, ultimately upregulating the expression of leukocyte adhesion molecules, including VCAM-1 and ICAM-1, as well as the chemokine CCL2 (Albarrán-Juárez et al., 2018). Disturbed flow generates low shear stress and OSS.

*In vitro* studies have shown that low shear stress activates the CX3CR1/NF-κB signaling axis, leading to the upregulation of pro-inflammatory cytokines (TNF-α and IL-6) (Wang et al., 2023b), adhesion molecules (VCAM-1, ICAM-1, and E-selectin), and the chemokine MCP-1 in endothelial cells. This promotes the adhesion and accumulation of neutrophils, monocytes, and T cells (Nordlohne and von Vietinghoff, 2019; Huang et al., 2023; Han et al., 2025).

OSS activates inflammatory pathways through mechanisms and downstream gene targets distinct from those induced by low shear stress (Chen et al., 2024). OSS is a pro-inflammatory biomechanical environment that drives an endothelial inflammatory phenotype. In this mechanical context, Piezo1 upregulates the NF-κB pathway component p65 and VCAM-1 expression, subsequently promoting monocyte adhesion to the endothelium (Davis et al., 2023). Specifically, *In vitro* studies have demonstrated that OSS upregulates the expression of bone morphogenetic protein 4 (BMP4) in endothelial cells, which subsequently activates NF-κB in a paracrine manner and increases ICAM-1 expression. In contrast, laminar shear stress downregulates BMP4 expression (Sorescu et al., 2003). BMP4 is also a mechanosensitive protein; its expression is regulated by the YAP/TAZ pathway and is associated with vascular calcification (Chandran Latha et al., 2021). NF-κB acts as an upstream transcriptional regulator of the NLRP3 inflammasome (Liu et al., 2022). It upregulates the expression of NLRP3 and caspase-1, thereby promoting the release of IL-1β (Fish and Kulkarni, 2024).

### 5.2 Laminar flow inhibits endothelial inflammation via Piezo1

#### 5.2.1 Piezo1-eNOS/NO signaling

*In vitro* and animal studies have shown that both laminar and disturbed flow activate the Piezo1/P2Y2/Gq/G11 pathway. However, unlike disturbed flow, LSS does not activate integrins. Instead, LSS activates the PI3K/AKT pathway, leading to phosphorylation of eNOS and sustained production of the vasodilator NO, thereby promoting vasodilation and exerting anti-inflammatory effects (Wang et al., 2016b; Albarrán-Juárez et al., 2018). In addition to the integrin-independent PI3K/AKT pathway, the Ca^2+^ signal induced by LSS can also activate AKT via the cAMP-IP3R2 pathway, thereby promoting eNOS phosphorylation and directional cell alignment (Nunez et al., 2023).

#### 5.2.2 Piezo1-KLF2/KLF4 signaling

Krüppel-like factor 2 (KLF2) and Krüppel-like factor 4 (KLF4) are endothelial transcription factors whose expression is induced by FSS in a dose-dependent manner (Lu et al., 2019), promote the anti-inflammatory and anti-thrombotic phenotypes of ECs (Zheng et al., 2022). Laminar shear stress activates Piezo1, leading to Ca^2+^ influx. This, in turn, triggers the MEKK3/MEK5/ERK5 signaling cascade via calmodulin-dependent protein kinase II (CaMKII), resulting in the upregulation of KLF2 and KLF4. The upregulated KLF2 and KLF4 then inhibit NF-κB activity (SenBanerjee et al., 2004) orchestrating anti-inflammatory, anti-thrombotic, and anti-oxidative gene programs. KLF2 promotes eNOS phosphorylation, promoting an anti-inflammatory endothelial phenotype, thereby maintaining vascular function and homeostasis (Zheng et al., 2022). *In vitro* studies have demonstrated that LSS upregulates KLF2 to maintain endothelial homeostasis, whereas OSS significantly downregulates KLF2 via the Piezo1-Ca^2+^-AP-1 pathway (Jieensi et al., 2026). However, low shear stress suppresses MEKK3/ERK5/KLF2 signaling, leading to nuclear translocation of Smad2/3 and the initiation of inward vascular remodeling (Deng et al., 2021).

In summary, different exercise modalities generate distinct FSS patterns, which may differentially regulate Piezo1 activation and downstream signaling networks. MICT and HIIT are generally considered to improve endothelial function. Short-and long-term MICT, as well as short-term HIIT, increase FSS amplitude and frequency in human, activate the PI3K/AKT pathway, enhance NO bioavailability, reduce ET-1 production, thence improve vascular tone and endothelial function (Li et al., 2023). As mentioned above, both resistance exercise, moderate intensity aerobic exercise, and HIIT impose high-amplitude laminar shear stress on the vessel wall, which may effectively activate the Piezo1-dependent eNOS/NO and KLF2/KLF4 pathways, thereby promoting an anti-inflammatory, vasoprotective endothelial phenotype and contributing to cardiovascular adaptation. In contrast, sustained high-intensity exercise can uncouple eNOS, further reducing NO production and increasing ET-1 levels. It also induces oxidative stress and pro-inflammatory responses, which may impair endothelial function (Li et al., 2023). This detrimental effect may be attributable to the OSS-induced by high-intensity aerobic exercise, which biases Piezo1 activation toward a pro-inflammatory endothelial phenotype. Further studies are required to investigate the dose-response relationship between exercise, blood flow patterns, inflammatory responses, and the specific Piezo1-dependent mechanisms involved (**Table 1**).

**Table 1:** An integrative framework linking exercise modalities to endothelial inflammatory outcomes via the FSS-Piezo1 axis.

Exercise modality	FSS characteristic	Downstream signaling pathways activated by FSS	Outcomes of endothelial cells	Reference
Moderate intensity aerobic exercise	laminar shear stress • Anterograde flow dominant • Unidirectional, with no retrograde component	• Piezo1 → eNOS/NO • Piezo1 → KLF2/KLF4	Anti-inflammation • VCAM-1/ICAM-1 ↓ • TNF-α/IL-6 ↓ • NF-κB ↓	(Robinson et al., 2017; Kim et al., 2024; Jieensi et al., 2026)
High intensity aerobic exercise	High amplitude laminar shear stress •Increased retrograde shear rate	Potential activation of pro-inflammatory pathways	FMD ↓	(Green et al., 2005; Birk et al., 2013)
HIIT	Pulsate laminar shear stress •High anterograde flow during exercise bouts • Brief retrograde flow during recovery periods	Remain inconsistent	• Pericyte coverage ↑ •vascular adaptation ↑	(Lyall et al., 2019; Maufroy et al., 2026b)
Resistance exercise	laminar shear stress • unidirection laminar shear stress predominates	• Piezo1 → eNOS/NO • Piezo1 → KLF2/KLF4	Anti-inflammation •No significant retrograde shear-associated inflammation	(Beech, 2018; Thomas et al., 2020)
Sedentary	Low shear stress (<4 dyn/cm²)	• CX3CR1/NF-κB ↑	Pro-inflammation • VCAM-1/ICAM-1 ↑ • TNF-α/IL-6 ↑	(Restaino et al., 2015; Wang et al., 2022a)

## 6 Conclusion and prospects

Piezo1 is a central mechanosensor through which endothelial cells perceive FSS. A limitation of current studies is that most evidence linking exercise directly to Piezo1 activation remains indirect. Future studies combining conditional Piezo1 knockout with well-controlled exercise paradigms are needed to establish causality. By modulating the magnitude and direction of FSS, exercise may differentially activate Piezo1, thereby bidirectionally regulating pro-inflammatory and anti-inflammatory pathways in the vascular endothelium. This mechanistic framework offers a novel mechanobiological perspective for understanding how exercise benefits cardiovascular health.

We have systematically demonstrated that physiological laminar shear stress activates Piezo1-dependent eNOS/NO and KLF2/KLF4 pathways to exert anti-inflammatory effects, whereas disturbed flow generates low or oscillatory shear stress triggers Piezo1-mediated YAP/TAZ and NF-κB signaling, promoting endothelial inflammation. Distinct exercise modalities, by generating specific FSS profiles, may determine the directionality of Piezo1-regulated inflammatory networks.

This framework has clear translational implications. Optimizing exercise type and intensity could be leveraged to directionally modulate Piezo1 activation. Moreover, Piezo1 itself represents a potential therapeutic target for shear stress-related vascular inflammation, and FSS patterns may serve as a quantifiable parameter for the design of future exercise prescriptions.

Despite considerable progress, several key issues remain. For instance, First, Piezo1-mediated mechanotransduction may exhibit vascular bed specificity. Future studies should systematically characterize Piezo1 expression, activation thresholds, and downstream signaling in different vascular beds to understand how exercise differentially affects distinct vessel types. Additionally, Piezo1, along with other structures such as the glycocalyx, cytoskeleton, and lipid rafts, forms a mechanosensory complex that synergistically senses fluid shear stress, However, the crosstalk between Piezo1 and other mechanosensitive channels and structures, such as TRPV4, remains to be elucidated. Third, understanding how FSS patterns shape Piezo1 signaling outcomes could inform the design of personalized exercise prescriptions. Future studies should define the dose-response relationship among exercise intensity, hemodynamic profiles, and Piezo1 activity, to optimize beneficial Piezo1-mediated vascular responses while minimizing excessive pro-inflammatory signaling, ultimately enabling the development of personalized exercise regimens based on individual cardiovascular risk profiles. Addressing these questions will provide a robust theoretical foundation for precisely targeted exercise interventions and Piezo1-based therapeutic strategies for cardiovascular diseases.
